# Cetuximab treatment in a patient with metastatic colorectal cancer and psoriasis

**DOI:** 10.3747/co.v15i4.228

**Published:** 2008-08

**Authors:** B. Neyns, V. Meert, F. Vandenbroucke

**Affiliations:** * Department of Medical Oncology, Universitair Ziekenhuis Brussel, Brussels, Belgium.; † Department of Pathology, Universitair Ziekenhuis Brussel, Brussels, Belgium.; ‡ Department of Radiology, Universitair Ziekenhuis Brussel, Brussels, Belgium.

**Keywords:** Cetuximab, psoriasis, colorectal cancer, skin toxicity

## Abstract

Cetuximab, a monoclonal antibody directed against the epidermal growth factor receptor, has activity against colorectal cancer. Treatment is associated with skin toxicity, and the safety of cetuximab in patients with psoriasis is unknown. We report the case of a male patient with stage iv colorectal cancer (crc) and a life-long history of extensive psoriasis. This patient experienced a durable remission of his crc and major improvement of his psoriasis during single-agent treatment with cetuximab. We conclude that, despite its known skin toxicity, cetuximab treatment can be offered to colorectal patients suffering from psoriasis.

## 1. CASE REPORT

Cetuximab, an immunoglobulin G1 monoclonal antibody directed against the human epidermal growth factor receptor (egfr), is effective for the treatment of metastatic colorectal cancer (crc). Few data on the safety of cetuximab in patients with pre-existing chronic skin diseases are available, but here, we report on an 80-year-old man, successfully treated with cetuximab, who had suffered from extensive treatment-resistant psoriasis since adolescence. Diverse lifelong treatments had provided only partial relief. Recently, he had used calcipotriol ointment up to 4 times weekly.

Our patient underwent resection of a well-differentiated adenocarcinoma (sigmoid, stage iii, pT4N1M0) in June 2003, followed by 8 months of adjuvant chemotherapy [bolus 5-fluorouracil (5-fu) and leucovorin (lv)]. In September 2004, bilateral inoperable liver metastases were treated with capecitabine until April 2005; best response was stable disease. At progression, second-line treatment was 5-fu/lv/irinotecan (folfiri) from September 2005 until May 2006, achieving a partial response. Disease progressed in August 2006, and lv/5-fu/oxaliplatin (folfox7) was administered until December 2007. Disease stabilized before progressing again.

**FIGURE 1:**
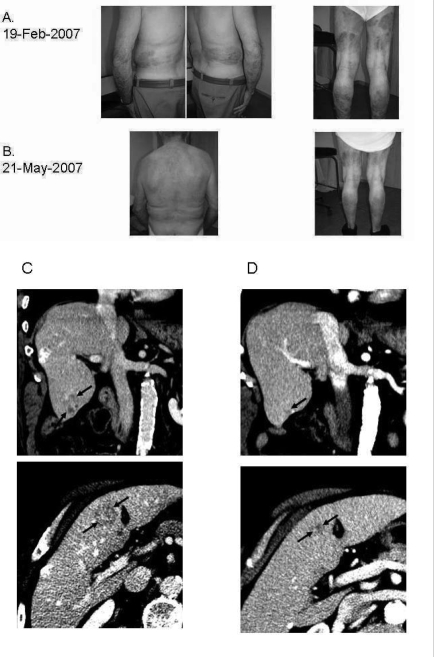
Tumour response and resolution of psoriasis in a patient with metastatic colorectal cancer (crc) treated with cetuximab monotherapy. An 80-year-old man was treated with cetuximab third-line for metastatic crc. His psoriasis on lower limbs, elbows, forearms, trunk, and face diminished in intensity after 6 weeks of treatment. This improvement was accompanied by almost complete regression of liver metastases. (A,C) February 2007. (B,D) May 2007.

After a short treatment-free interval, cetuximab monotherapy (400 mg/m^2^, intravenously over 2 hours on day 1, and 250 mg/m^2^, intravenously over 1 hour on day 8 and once weekly thereafter) was initiated in February 2007. Immunohistochemistry for egfr on the primary tumour was negative. Over the next 6 weeks, the psoriasis lesions lessened considerably (Figure 1), and the patient reduced calcipotriol ointment applications to once weekly.

The main adverse effects of cetuximab are dermatologic. Grade 2 dermatitis and folliculitis developed in this patient, but were controlled by topical clindamycin and then lessened in intensity. On re-evaluation in May 2007, almost complete regression of the liver metastases and a normalization of blood concentrations of carcinogenic embryonic antigen were observed. Throughout this period, no local or systemic corticosteroids were administered. In September 2007, irinotecan was added to cetuximab treatment because of disease progression. Disease control was regained, and the patient is still receiving cetuximab (July 1, 2008). He also continues to experience a marked improvement of his psoriasis.

## 2. DISCUSSION AND CONCLUSIONS

We believe that this is the first case report of the safety of cetuximab monotherapy in a patient with metastatic and psoriasis. Trivin *et al.* 1 crc reported on a patient who had both a tumour response and durable remission of psoriasis after treatment with cetuximab in combination with folfiri. However, that case did not differentiate between a potential beneficial effect of the chemotherapy and the effect of the cetuximab.

Durable remissions of psoriasis have also been reported in patients undergoing high-dose chemotherapy for hematologic malignancies 2–4.

We conclude that a previous history of psoriasis or active lesions should not be regarded as a contraindication for cetuximab treatment in patients with advanced crc. Psoriasis is associated with abnormal expression of egfr in the involved skin 5. It is possible that cetuximab may induce remission of psoriasis through regulation of this abnormal egfr metabolism.
